# Real-Time Size and Mass Estimation of Slender Axi-Symmetric Fruit/Vegetable Using a Single Top View Image

**DOI:** 10.3390/s20185406

**Published:** 2020-09-21

**Authors:** Tri Huynh, Ly Tran, Son Dao

**Affiliations:** 1 School of Electrical Engineering, International University, Vietnam National University HCMC, Ho Chi Minh City 700000, Vietnam; trihuynh6796@gmail.com; 2 School of Industrial Engineering and Management, International University, Vietnam National University HCMC, Ho Chi Minh City 700000, Vietnam; tvly@hcmiu.edu.vn

**Keywords:** volume estimation, mass estimation, image processing, machine vision, vegetable, water displacement method

## Abstract

Among the physical attributes of agricultural materials, mass, volume, and sizes have always been important quality parameters. Previous research focused mostly on volume estimation using stereo-based approaches, which rely on manual intervention or require a multiple-cameras set up or multiple-frames captures from different viewing angles to reconstruct the three-dimensional point-cloud information. These approaches are tedious and not suitable for practical machine vision systems. In this work, we only use a single camera mounted on the ceiling of the imaging chamber, which is directly above the fruit/vegetable to capture its top-view, two-dimensional image. We developed a method to estimate the mass/volume of agricultural products with axi-symmetrical shapes such as a carrot or a cucumber. The mass/volume is estimated as the sum of smaller standard blocks, such as chopped pyramids, an elliptical cone, or a conical cone. The computed mass/volume showed good agreement with analytical and experimental results. The weight estimation error is 95% for the case of the carrot and 96.7% for the cucumber. The method proved to be sufficiently accurate, easy to use, and rotationally invariant.

## 1. Introduction

With the increase of the population and the improvement of life from day to day, people’s demands on quality, quantity, and type are getting higher. Therefore, factories must expand and change to follow up the amount of production. Currently, people are researching how to speed up the production line but maintain the same quality of food.

Size, weight, and shape are the major indices in determining standards in the industry such as packaging, grading, processing, and conveying. This makes researchers have to investigate the physical characteristics of fruit and vegetables to find the solution boosting up the production line [1,2,3,4,5,6,7,8,9,10]. There are some works that classified the fruit/vegetables based on its shape and features. They applied various classifiers (Artificial Neural Network (ANN), Fourier-descriptor (FD), …) for some types of fruit/vegetable listed in Table A1 of Appendix A. In addition, there is a superior survey that delivers not only a summary of many different methods, tasks, and concepts but also the key challenges and future directions for food computing [11]. Typically, the mass of the product will be measured using a load cell incorporated with a conveyor belt such as C35 AdvancedLine WD Checkweigher by Mettler Toledo, which can measure weight from 25 g to 7000 g with throughput up to 250 packets per minute or 2000 g Checkweigher from A&D Inspection has throughput up to 320 packets per minute or digital scale for individuals or businesses [12,13]. The load cell converts gravitational force into a measurable electrical signal. Usual load cell requires filters and Analog-to-digital Converter (ADC) to work. This leads to a high mechanical structural cost and is also time-consuming when we have to recalibrate the systems after a certain amount of usages time depending on the hardware quality.

Due to the limitation of the load cell, many researchers try to find other methods to estimate weight/volume without using a load cell [14,15,16,17,18,19,20,21,22,23,24,25,26,27,28,29,30,31,32,33,34,35,36,37,38]. One of the most accurate ways is to estimate the volume of the product and using a pre-determined “density” to calculate the actual weight of the object because mass and volume are correlated to a certain degree. For instance, the correlation coefficient (R^2^) between mass and volume of mango is 0.9978 [15], tangerine, lime, lemon, and orange are 0.9045, 0.9592, 0.9641, and 0.9232, respectively [28].

The previous method to estimate the volume of an object is the water displacement method (WDM), which is presented in References [16,17]. The water displacement method is known to give an accurate result by ensuring the water fully submerges the objects. However, this method is hard to do by always keeping the buoyancy force of water acting on the objects. In addition, it is difficult to maintain the force so that the object remains afloat in the water as well as get a good reading.

Another method is a 3D reconstruction using 3D scanning from multiple 3D views [22,23,24,25,26,27,39]. The 3D construction returns highly accurate results, but it requires many cameras. Moreover, the calibration between cameras and the perfect timing when capturing the images at the same time is very complex. It also requires the complicated algorithm to preprocess and reconstruct a 3D representation of the object. On the other hand, the 3D scanning is only applicable in the laboratory because we have to work many times at many different angles. Therefore, the 3D point clouds reconstruction is very time-consuming.

Some researchers also used geometrical features of an object to estimate volume using Multiple Linear Regression (MLR) [14]. The MLR is the supervised learning with a procedure called Ordinary Least Square [40,41]. It is used to predict which one has two or more independent inputs (the widths and volume of an object) and return the function as below.

(1) $$y=b_{0}+\sum_{i=1}^{n}b_{i}x_{i},$$

We have, in the total of n inputs, $b_{i}$ is the coefficient of a set of chosen features $x_{i}$, and y is the predicted volume.

In addition, the Artificial Neural Network (ANN) has also been used for volume estimation [14,26,37]. One of the most popular ways to estimate the volume is to use a single image and measure the widths and/or lengths to feed into their model [14,37]. There are many types of ANNs in the literature [26,37,42]. However, the Levenberg-Marquardt algorithm (LM) still provides sufficiently accurate results in volume estimation [26,37]. The Levenberg-Marquardt neural networks were used to form non-linear combinations of various features to estimate fruits’ volumes [37,42]. It takes the sum of squared of nonlinear functions to get the minimum of a function. With its simple and robust platform, we decided to apply it to predict the volume of fruits/vegetables.

(2) $$(J_{k}^{T}J_{k}+\lambda_{k}I)p_{k}=-J_{k}^{T}f_{k},$$

In Equation (3), J is defined as the Jacobian matrix, $\lambda_{k}$ is a non-negative scalar, and *I* is the identity matrix. For some scalar Δ related to $\lambda_{k}$, the vector $p_{k}$ is the solution of:

(3) $$\underset{p\in R^{n}}{minimize}\;\frac{1}{2}{\left\|J_{k}p+f_{k}\right\|}_{2}^{2},$$

(4) $$subject\;to\;{\left\|p\right\|}_{2}<\Delta ,$$

Even though the accuracy of ANNs is not high and each approach works for one type of product only, those are very promising approaches. If we can find the appropriate feature, the accuracy will increase so it will be more suitable for real-time implementation.

**Figure 1:**
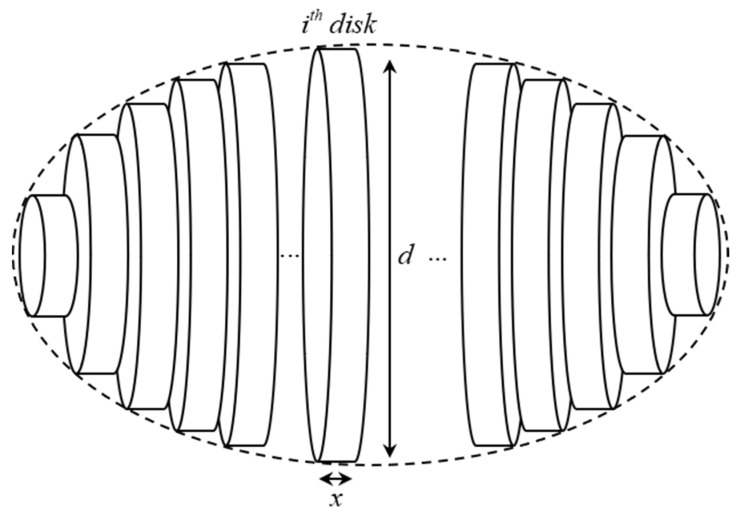
An example object is sliced into many disks.

On the other hand, there are some proposed that used only one two-dimensional image of an object to estimate its volume by using the disk method [29,30,31,32,33]. They slice the fruit into many disks with the properties shown in Figure 1 [15,28,29,30,31,32,33].

Figure 1 shows that, once we have the dimension (d) and height (x) of each slice, we can calculate its volume (see Equation (6)) via its area (see Equation (5)). Lastly, the volume of an object is calculated by summing all the volume of *n* disks (see Equation (7)). In detail, the height (x) will equal 1 pixel to have the most precise estimated volume.

(5) $$A_{i}=\pi {\left(\frac{d_{i}}{2}\right)}^{2},$$

(6) $$V_{i}=A_{i}x,$$

(7) $$V=\sum_{i=1}^{n}V_{i},$$

Ibrahim et al. [15] also modified the disk method to fit with unpredictably shaped objects. Once they have two view images (top and side) of a mango, the area of each disk is defined in Equation (8).

(8) $$A_{i}=\pi \frac{d_{i}z_{i}}{4},$$

**Figure 2:**
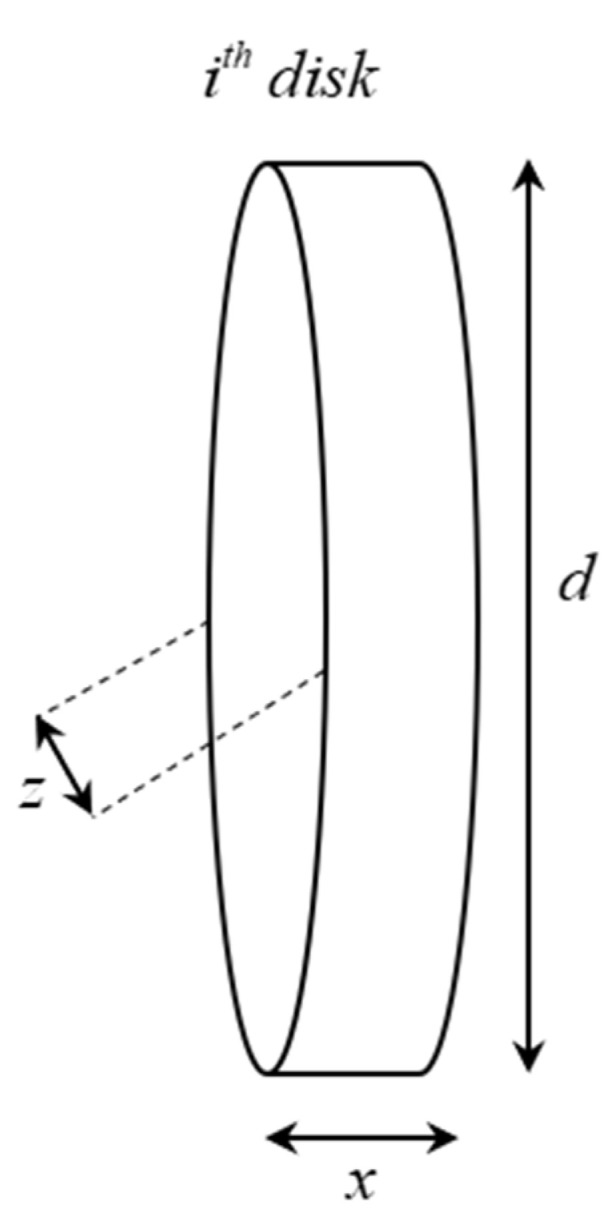
An example of a slice’s volume with two widths z and d, with z being the width observed from the top view and d being the width from the side view.

With $z_{i}$ and $d_{i}$ is the width of the *i*th disk from the top view and side view, respectively. Figure 2 illustrates one slice with the mentioned parameters in Equation (8).

The disk method, however, is one of the most suitable ones for objects that has a round or elliptical shape. With a fruit/vegetable shape like a carrot, it cannot return the highest results. Moreover, Venkatesh et al. researched that the tilt angle of the fruit or vegetable must be below 10 degrees for the reasonable volume estimation [19]. Therefore, the alignment step must be integrated into the production line. This also increases the cost of the alignment module or workforce.

Some fruits/vegetables also have an elliptical cone or cap sphere shape on its top or bottom such as a cucumber, melon, pumpkin, carrot, grape, tomato, kiwi fruit, passionfruit, etc. Among these fruits/vegetables, a carrot, a cucumber, a daikon, etc. are considered to be the slender axi-symmetric fruits or vegetables due to their height being much longer than their width. With this property, Vankatesh et al. cannot assume those fruits as an ellipsoid or a sphere [19]. In addition, this approach requires multiple manual captures with different angles. Thus, it is not practical to implement in a read machine vision system.

Our approach focuses on improving the accuracy and computational time of the disk method [29,30,31,32,33], so that the model will be accurate enough and fast for real-time measurement [36]. With only one top view image of the fruit or vegetable, we slice the object into a number of equal slices. Then, the volumes of the top and bottom parts are assumed to be the volumes of the elliptical cone. We considered each slice of the middle part as a chopped pyramid. Based on the results of those previously mentioned steps, we can estimate the fruit/vegetable volume. In addition, since we know there is a high correlation between fruit/vegetable weight and its volume [15,19,28], we can infer the mass based on volume information. Once we get the volume of the top, the middle, and the bottom parts, we estimate the volume and the density, from which we can calculate the object’s weight. In this paper, we focus on the carrot and cucumber, which are ranked as the most common vegetables in the world [43].

We divided the content of this paper into three main sections. In the introduction, we briefly mention the current concerns regarding volume measurement of the fruit/vegetable in the assembly lines as well as in the laboratory settings. In addition, the literature review that we are going to apply and compare is also discussed in this section. The Materials and Methods section presents our proposed approach and how we collect data. The result shows the outcome of our method and other research studies and compare each.

## 2. Materials and Methods

### 2.1. Materials

In this research, both 191 carrot and 160 cucumber samples were chosen randomly from the supermarket after they were harvested for two to three days.

**Figure 3:**
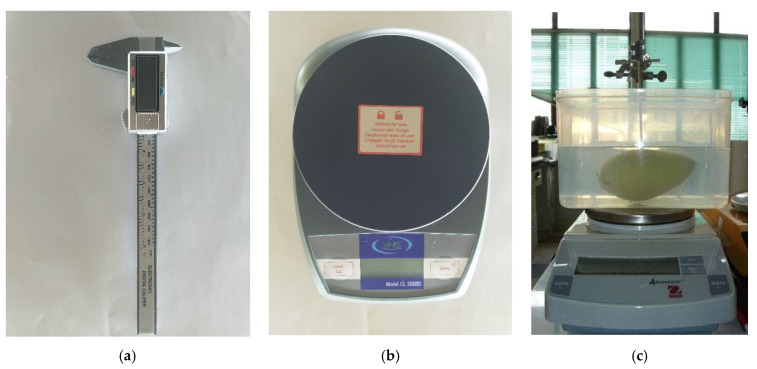
Measurement tools: (**a**) digital caliper, (**b**) digital scale, and (**c**) water displacement method [39].

For the reference length and width of the fruit/vegetable, we used the digital caliper with an error of ±0.1 mm, as shown in Figure 3a. Figure 3b reveals the digital scale model VNS CL 2001 (division of 0.1 g and capacity of 2000 g) that we used to get the reference mass. To get the volume of the fruit or vegetable, we applied the water displacement method, which consists of a digital scale, a tank of water, and a stick to hold the fruit/vegetable while being fully submerged. This is similar to the work of Chalidabhongse et al., which is shown in Figure 3c.

Various instruments were used to measure fruits’ physical properties. Length, width, and thickness are measured using a Vernier caliper. The fruits’ actual volume is measured by the water displacement method. In this method, fruits were submerged in distilled water. We can obtain the volume of the fruit as a function of three kinds of mass: (1) The mass of the measurement apparatus with water filled to the marking level, (2) mass of the fruit, and (3) the mass of the apparatus with the fruit submerged with the same water level as in (1).

Fruit volume, *V*, is then given by the equation below with $M_{1},M_{2},M_{3}$ being the three mentioned masses. These are actually the weight of the water displaced by the fruit and $\rho_{water}$ is the density of the water.

(9) $$V=\frac{M_{1}+M_{2}-M_{3}}{\rho_{water}}$$

Forbes et al. [14] improved the original WDM by integrating a funnel-like tube on top of a wide water container. The funnel-like tube helps to prevent the fruit from floating to the surface, which will reduce the measurement’s accuracy. The narrow tube on top reduces the measurement error of the setup since only a small change in water volume results in a larger change in the water level within the narrow tube.

### 2.2. Our Proposal

**Figure 4:**
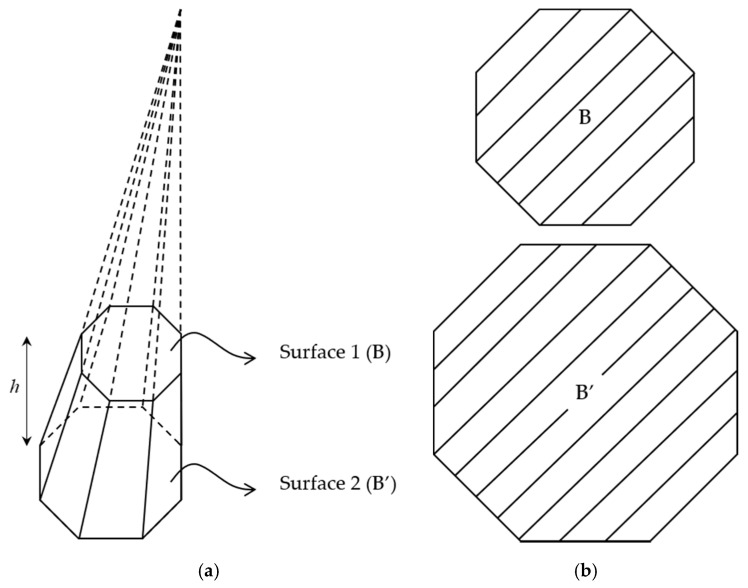
Pyramid: (**a**) Chopped pyramid and (**b**) cross-section of two surfaces *B* and *B*’.

With the slender axi-symmetric property of fruits/vegetables, we slice the fruits/vegetables into many parallel parts. For each part, Figure 4 and Equation (10) demonstrates an example and formula to calculate its volume, respectively.

Once we have the area of two area *B* and *B*′ ($\frac{B}{B^{\prime }}=$ constant) of a chopped pyramid, its volume is defined as below.

(10) $$V_{chopped-pyramid}=\frac{h}{3}\left(B+B{}^{\prime }+\sqrt{BB{}^{\prime }}\right),$$

#### 2.2.1. Volume of the Cross-Section of the Circular Vegetable or Fruit

For the fruit that has a round shape, its cross-section is also a circle such as lemons, tangerines, clementine, orange, etc. Therefore, the area *B* and *B*′ in Equation (10) are now defined as Equations (11) and (12) with r and $r{}^{\prime }$ being the radius of the top and the radius of the bottom of the cross-section.

(11) $$B=\pi r^{2},$$

(12) $$B{}^{\prime }=\pi r^{\prime 2},$$

#### 2.2.2. Volume of the Cross-Section of an Elliptical Vegetable or Fruit

**Figure 5:**
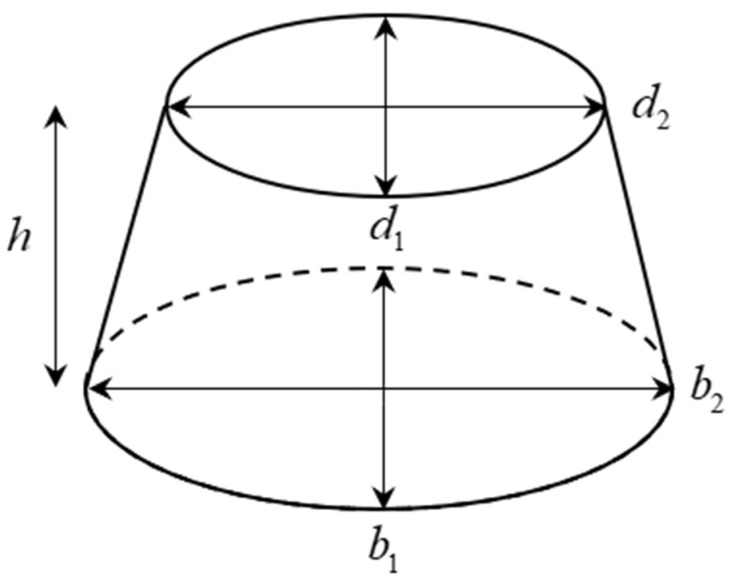
Cross-section of an elliptical vegetable or fruit.

Figure 5 illustrates one cross-section (frustum) of an elliptical vegetable or fruit and Equations (13) and (14) show the formulas of calculating the areas *B* and *B*′.

(13) $$B=\pi \left(\frac{d_{1}}{2}\right)\left(\frac{d_{2}}{2}\right),$$

(14) $$B{}^{\prime }=\pi \left(\frac{b_{1}}{2}\right)\left(\frac{b_{2}}{2}\right),$$

#### 2.2.3. Volume of the Cross-Section of an Arbitrary Polygon Vegetable or Fruit

For the fruit with a star shape such as a starfruit or bell peppers or having a complicated shape, the area *B* and *B′* can be derived when analyzing its physical properties or approximate its shapes/cross-section as one of the common geometry forms.

#### 2.2.4. Volume of the Top and Bottom of a Circular or Elliptical Vegetable or Fruit

**Figure 6:**
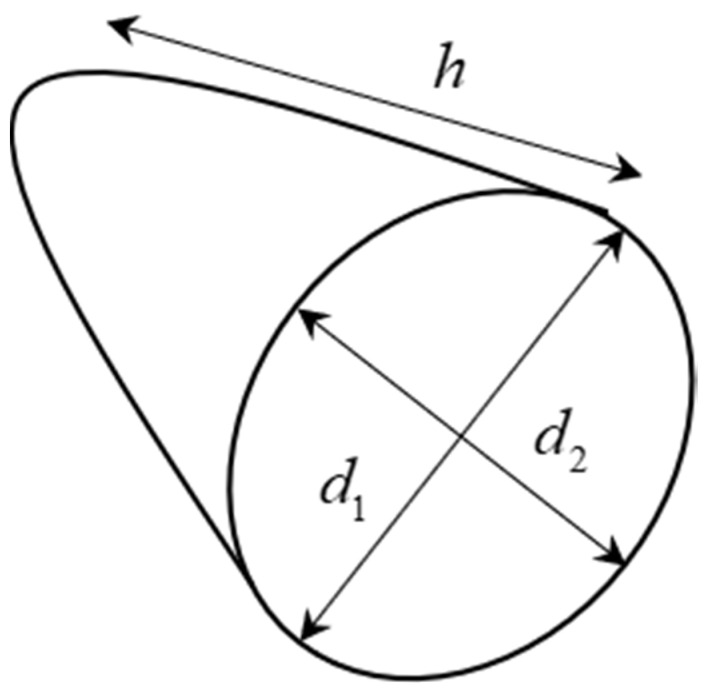
Example of the top and base part of an elliptical vegetable or fruit.

The top and bottom of these vines and underground fruits/vegetables are special with an elliptical cone shape (see Figure 6 and Figure 9a). Therefore, the volume top and bottom part can be estimated by using the elliptical cone formula as detailed in Equation (15).

(15) $$V_{top/base}=\frac{\pi \times d_{1}\times d_{2}\times h}{6},$$

With all the chopped-pyramid volumes of the body and two elliptical cone volumes of the top and bottom of the fruit/vegetable, we can estimate the total volume by using Equation (16).

(16) $$V_{total}=\sum V_{chopped-pyramid}+V_{top}+V_{base},$$

### 2.3. System Flow

**Figure 7:**
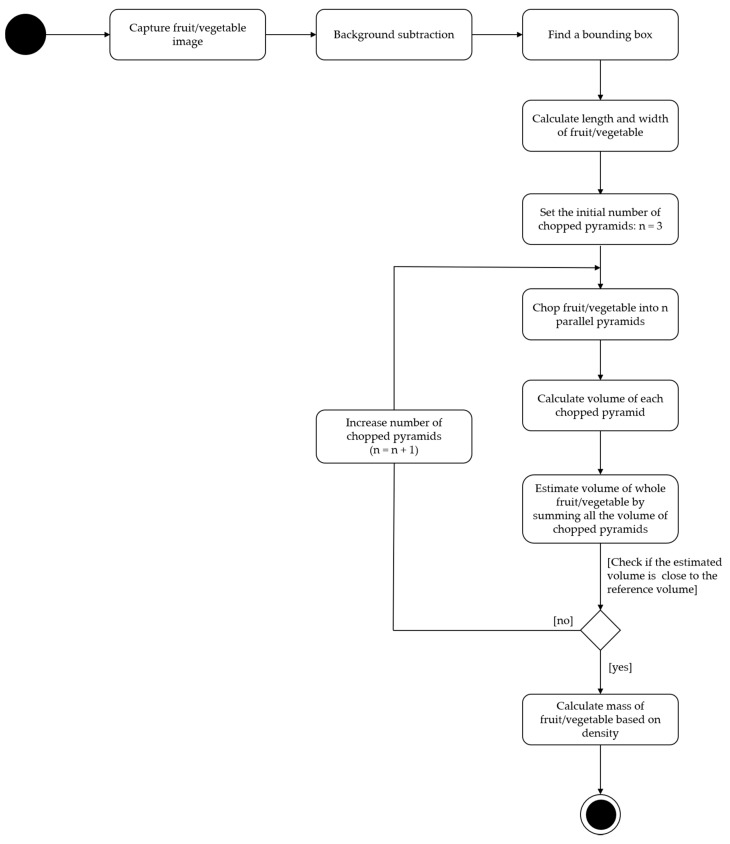
The activity diagram of the proposed system.

All the steps of our system are shown in Figure 7. The details of each step are described from Section 2.3.1, Section 2.3.2, Section 2.3.3, Section 2.3.4, Section 2.3.5 and Section 2.3.6 below.

#### 2.3.1. Hardware Setup

**Figure 8:**
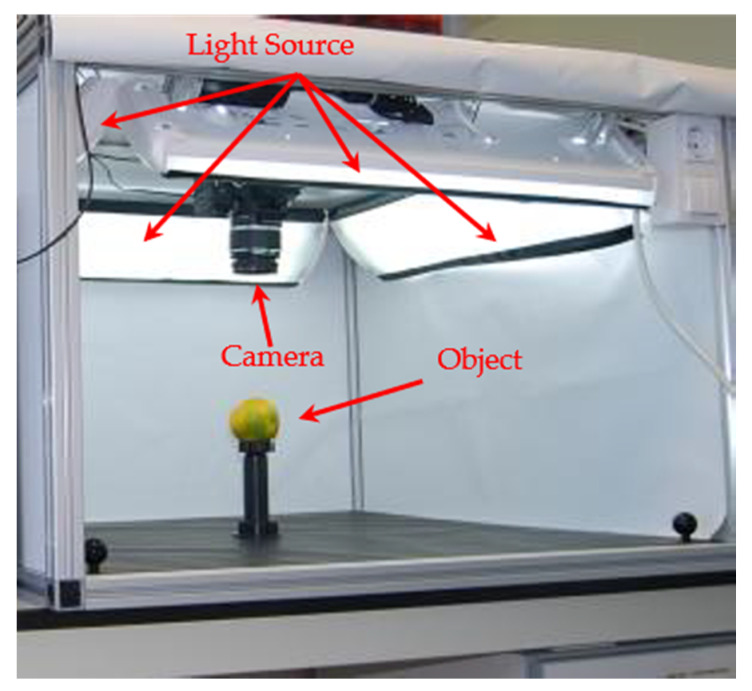
Schematic of the chamber experiment set-up.

Figure 8 displays that our system consists of an aluminum frame and one camera model CANON 60D (lens Tamron 17–50 mm, f2.8) mounted on top where it is perpendicular to the bottom of the frame. In addition, we placed a light source inside the frame to get rid of the shadow of an object. After the fruit or vegetable is captured, the system is then calibrated to have the pixel per centimeter square of the image.

#### 2.3.2. Background Subtraction

**Figure 9:**
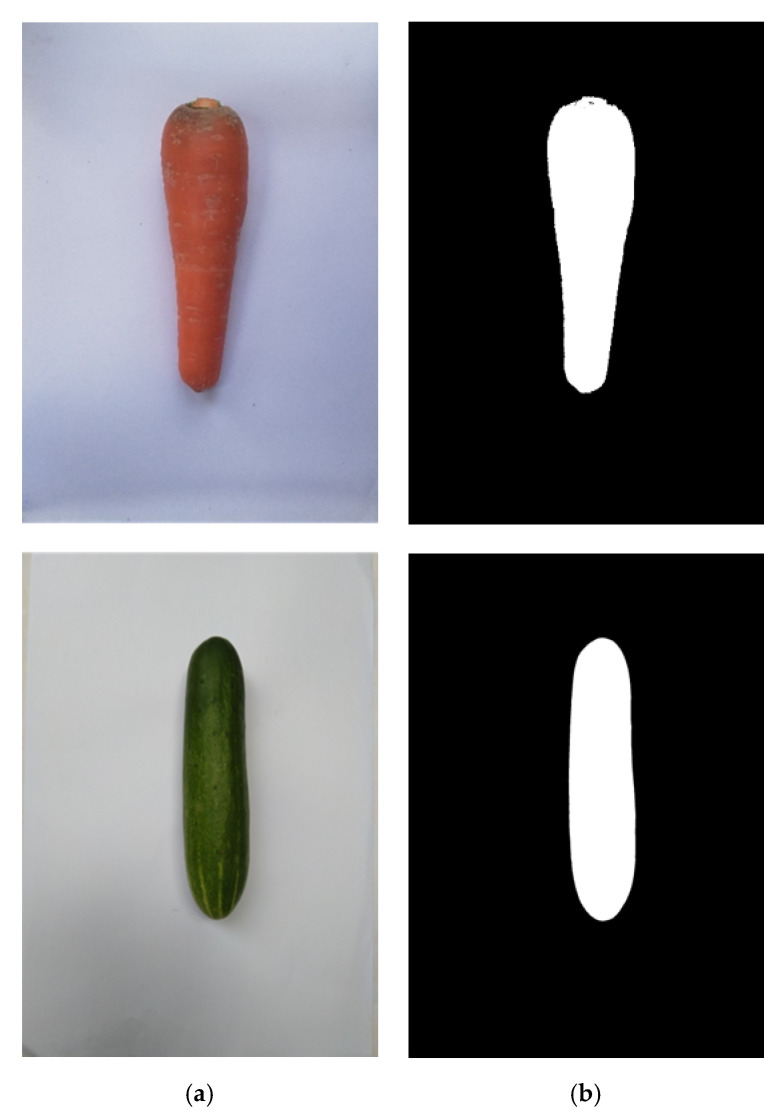
Background subtraction using image thresholding of the carrot and cucumber. (**a**) Original images and (**b**) images after thresholding.

There are many types of thresholds. Red-Green-Blue (RGB), Hue-Saturation-Value (HSV), or Hue-Saturation-Lightness (HSL) thresholding and Otsu’s method are one of the most famous in digital signal processing. This depends on the situation where researchers will apply the suitable one for their works. For instance, we extracted the red color and green color out of the white background, as illustrated in Figure 9. In addition, all the input images we used in this paper have a JPG format.

#### 2.3.3. Finding a Bounding Box

**Figure 10:**
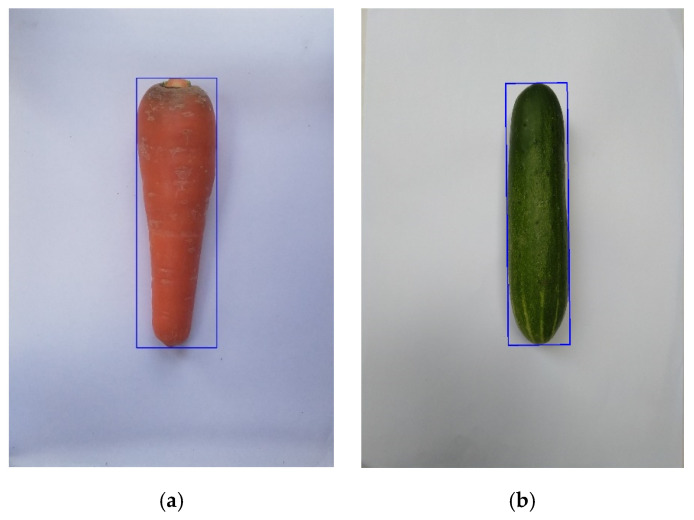
Bounding box (blue rectangle) in: (**a**) a carrot and (**b**) a cucumber.

The minimal bounding box technique supported by OpenCV will place a minimum rectangular box that fits the object in the image. The system will return four coordinates of the corners of the box by combining with the pixel per centimeter we have already calibrated and measured in Section 2.3.1. We can calculate the length as well as the maximum width of some fruits. Figure 10 illustrates that the object is inside a bounding rectangle (blue outlines) with a perfect fit. This step will be the premise to help reduce the time and increase the accuracy of the system.

**Figure 11:**
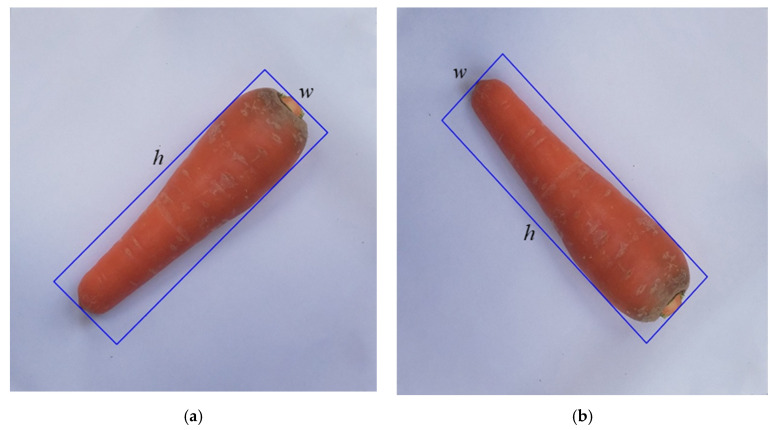
Bounding box on a carrot rotated in different angles. (**a**) 45° and (**b**) 135°.

By using the bounding box supported by the OpenCV library, the width *w* and height *h* of the box are constants on different angles. We can estimate the volume of the fruit or vegetable no matter how the object is rotated. Figure 11 shows that this technique can also align with the fruit/vegetable. This helps us reduce the alignment step in the production line.

#### 2.3.4. Approximating the Cross-Section as Known Geometrical Shapes

By using Equation (16), the major part of the volume calculation is the volume of the fruit/vegetable comprised of multiple chopped pyramids. Each chopped pyramid is assumed to have the same kind of cross-section even though their sizes can be different. In order to get this information, we take many cross-section samples and find the curve that deviates the least from a set of common curves, such as a circle, ellipse, etc.

**Figure 12:**
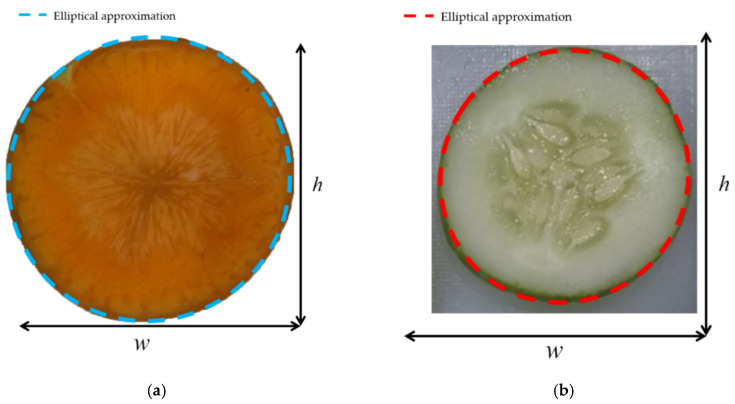
Elliptical representation of the cross-section of: (**a**) a carrot and (**b**) a cucumber.

The carrot and cucumber have very good elliptical curve fitting results, as shown in Figure 12. The ellipse uses the following equation.

(17) $$ax^{2}+bxy+cy^{2}+dx+ey+f=0,$$

We found that the average width/height ratios of the carrot and cucumber are 0.9531 and 0.9677, respectively.

#### 2.3.5. Volume Estimation

After all preprocessing and setup steps meet our criteria, the volume estimation step is specifically described in this section.

**Figure 13:**
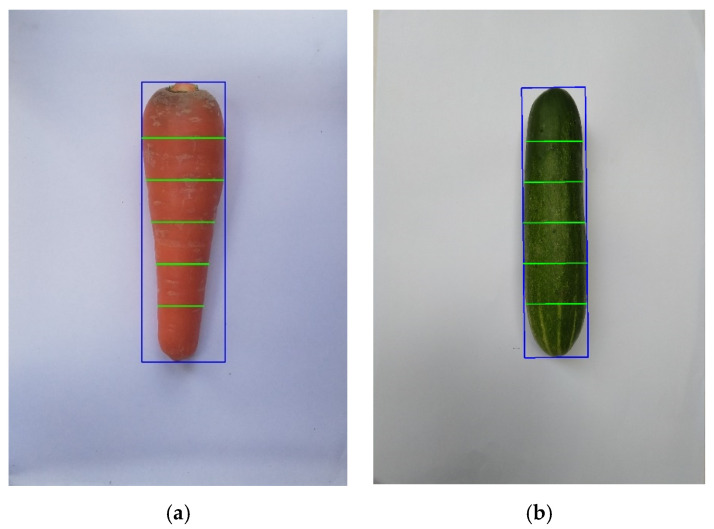
An example of a fruit or vegetable is sliced into six green equal slices: (**a**) carrot and (**b**) cucumber.

Since we can see that the fruits or vegetables related to a banana or an axi-symmetric one like a carrot, cucumber, white turnip, etc. has virtually round or ellipse cross-sections, we can split it into many single slices to easily estimate the volume of the fruit/vegetable. We then divided the fruit/vegetable into equal parallel slices, as illustrated in green lines in Figure 13. Each line has the same slope as the slope of the width of the bounding box.

By computing each chopped pyramid volume in the mid-regions and elliptical cone volume for the top and bottom part of the fruit/vegetable, as introduced in Section 2.2, the total volume of each experimental object can be estimated using Equation (16).

#### 2.3.6. Mass Estimation

**Figure 14:**
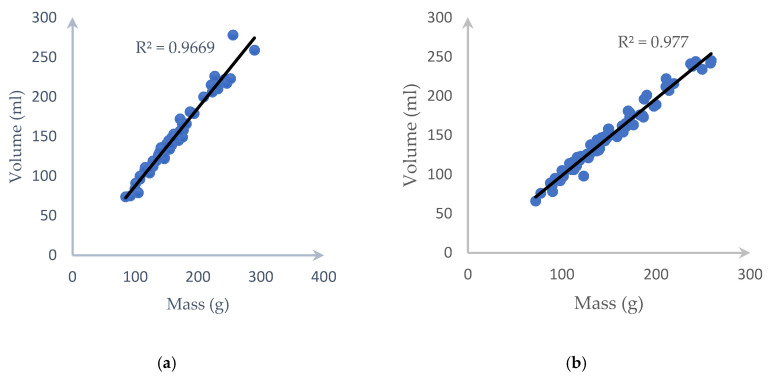
*R*^2^ and correlation between the mass measured by the digital scale and volume measured by the water displacement method for (**a**) a carrot and (**b**) a cucumber.

Figure 14 shows the weight against volume when using the water displacement method. We split our dataset into 70 samples for calculating the average density and the others for testing. Once the average density of each type of fruit/vegetable is calculated and combined with the estimated volume, we can get the estimated mass based on Equation (18) below.

(18) $$M_{estimated}=V_{estimated}\times d_{average},$$

## 3. Results

### 3.1. Proposed Method

**Figure 15:**
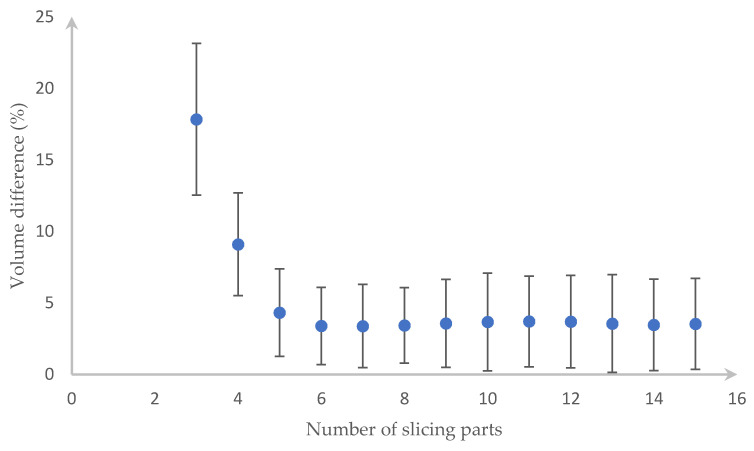
Percentage errors when slicing the carrot into 3 to 15 parts.

**Figure 16:**
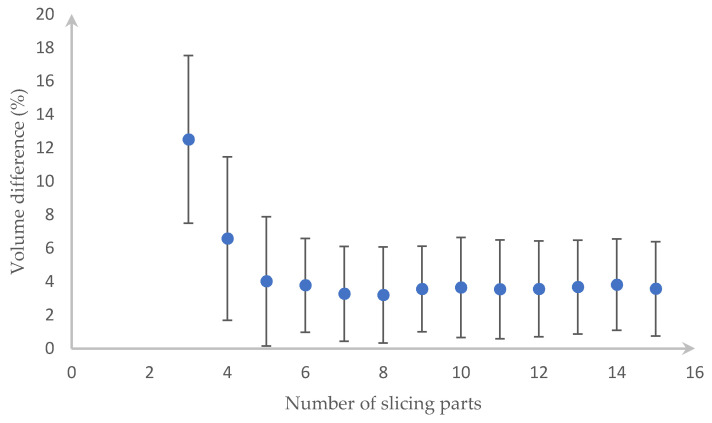
Percentage error when slicing the cucumber into 3 to 15 parts.

We have to divide each type of fruit/vegetable into different equal slices to get the most accurate value. After slicing the fruit/vegetable from three slices, we noticed that the errors are unvarying from the 13th slices. Because of this result, Figure 15 and Figure 16 shows the outcome of up to 15 slices with the error bar being the standard deviation of a different volume. In detail, those figures show that the carrot needs eight slices and the cucumber needs eight slices for the lowest error, which is 3.421% and 3.2025%, respectively.

**Figure 17:**
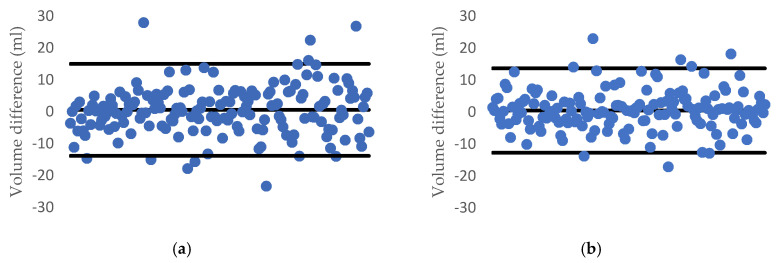
1.96-σ plot for the volume difference between the water displacement and our method: (**a**) a carrot and (**b**) a cucumber.

Over the dataset of 191 carrots and 160 cucumbers, the deviation plot of each fruit/vegetable when applying the proposed method is listed in Figure 17. We can see that almost all the estimated data are in the range of a 95% limit of agreement (outer lines) and the average difference between the two methods is the centerline.

**Figure 18:**
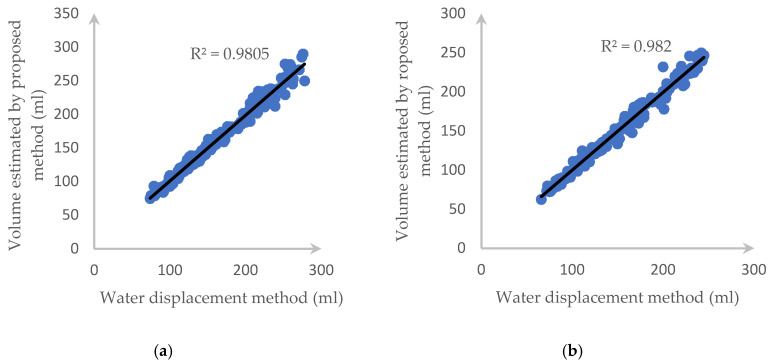
*R*^2^ and the correlation between the proposed method and the water displacement method for (**a**) a carrot and (**b**) a cucumber.

Figure 18 also illustrates the *R*^2^ value and the correlation of each tested fruit with our proposed method and water displacement and we can clearly see that our approach returns highly accurate results.

Table 1 shows the results of the paired t-test between our proposal and the water displacement method. The paired mean difference of the carrot is m = 0.6901 mL and the standard deviation is σ = 7.4791 mL. The paired mean difference and the standard deviation of the cucumber is m = 0.4436 mL and σ = 6.729 mL, respectively. The 95% confidence interval includes zero: a zero mean difference is well within the range of likely population outcomes. The mean difference between our proposed method and the water displacement method is not statistically significant at α = 0.05. This is because *p* > 0.05.

We also compare our research with other methods such as the disk method, the ANN method, and MLR and have some significant results.

### 3.2. Disk Method

**Figure 19:**
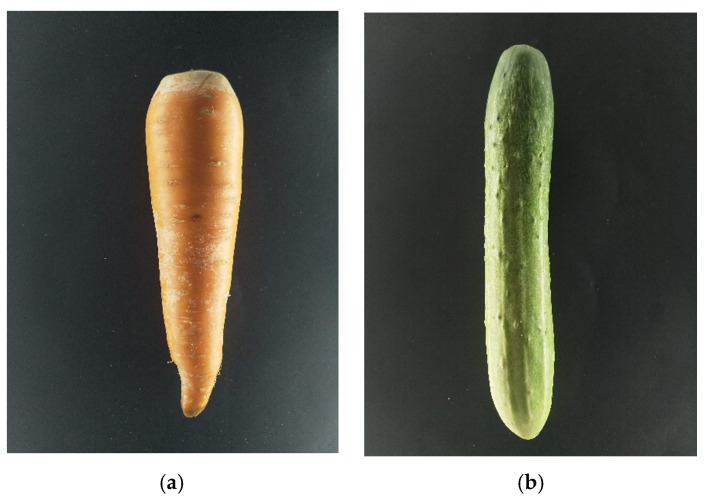
Top view images: (**a**) carrot, (**b**) cucumber.

**Figure 20:**
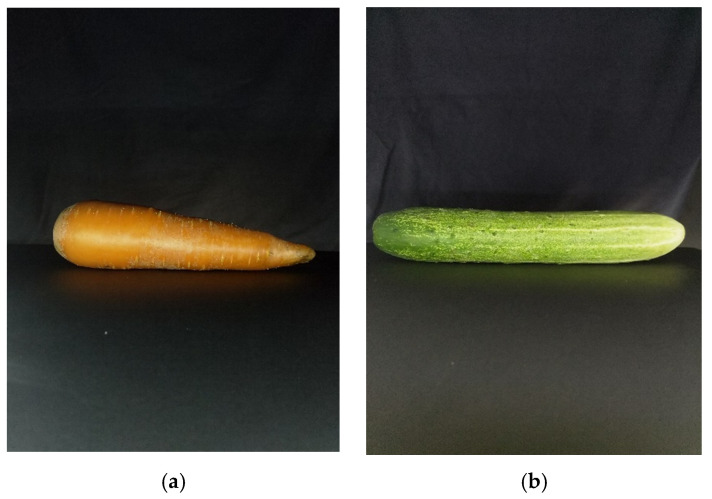
Side view images: (**a**) carrot, (**b**) cucumber.

As describe by Ibrahim et al. [15], we capture two sides of the fruits/vegetables. We placed one camera on the top and one on the side of the fruit/vegetable. Those cameras have the same specification and have been calibrated. Figure 19 shows the top view of the fruit/vegetable while Figure 20 shows the side view.

Next, their background was filtered out to get only the inner object as Section 2.3.2. After estimating all the dimensions of the top view and side view, we then applied Equation (8) to get the area of each slice. Once we had the areas, Equations (6) and (7) had been used to estimate the volume of the object.

**Figure 21:**
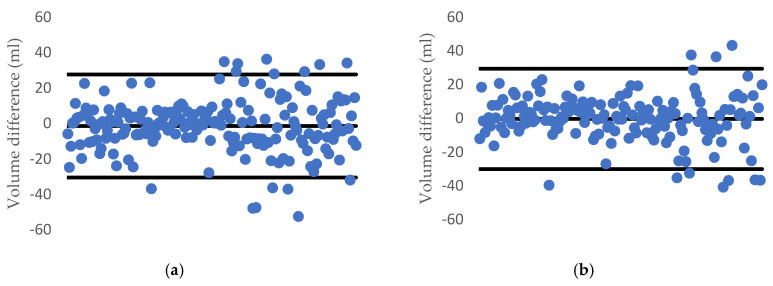
1.96-σ plot for the volume difference between the water displacement and Ibrahim’s method: (**a**) carrot and (**b**) cucumber.

**Figure 22:**
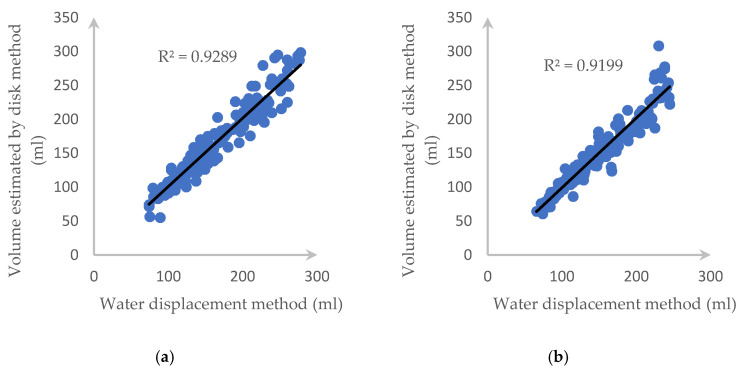
*R*^2^ and correlation between two side views using the disk method and the water displacement method for (**a**) a carrot and (**b**) a cucumber.

Figure 21 and Figure 22 describe the deviation of volume difference and correlation when using the disk method proposed by Ibrahim [15].

### 3.3. ANN Method

Örnek’s work is using a 1-layer ANN and the training algorithms that he used are Back Propagation (BP), Levenberg-Marquardt (LM), and Product Unit Neural Network (PUNN) with the number of neurons being 15, 14, and 2, respectively [37]. The outcomes are also potential, which is listed in Table 2.

**Figure 23:**
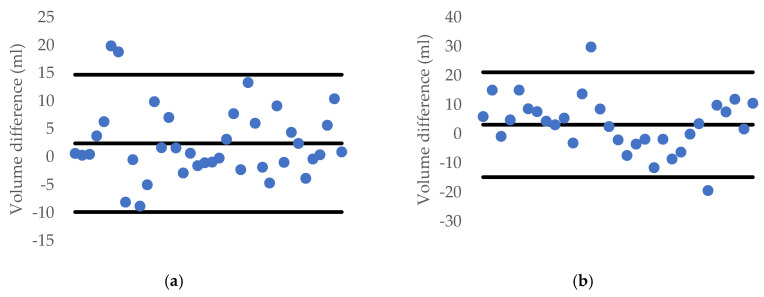
1.96-σ plot for the volume difference between water displacement and the ANN method: (**a**) carrot and (**b**) cucumber.

**Figure 24:**
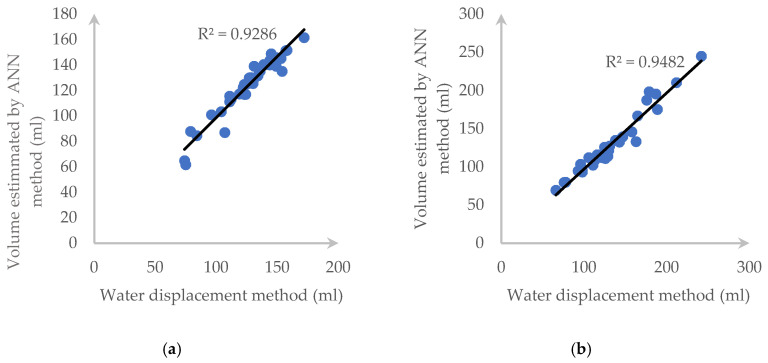
*R*^2^ and correlation between ANN and the water displacement method for: (**a**) a carrot and (**b**) a cucumber.

In this section, we reproduced Örnek’s works with the same setting but using our dataset. Each carrot and cucumber dataset is divided into two parts, which are the training set (153 carrot samples and 128 cucumber samples) and the testing set (38 carrot samples and 32 cucumber samples). The LM method and PUNN has the same result. Therefore, we just pick only the LM algorithm to test. In addition, we use the MATLAB R2019a software to perform this task. Furthermore, a 10-fold cross validation deviation of volume difference has been recorded in Figure 23. Moreover, Figure 24 shows the correlation of this method when compared with the water displacement method.

### 3.4. Using MLR

The result in Section 3.3 shows that there is a high correlation among length, widths, and volume of the carrot and cucumber. Due to that fact, in this section, we also apply the Multiple Linear Regression method to estimate the volume. The input is the same as the Örnek’s work with the input parameters being length, five parallel widths, and volume of each fruit or vegetable [37].

**Figure 25:**
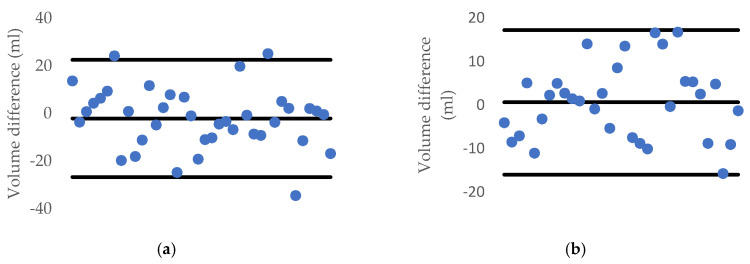
1.96-σ plot for the volume difference between the water displacement and MLR method: (**a**) carrot and (**b**) cucumber.

**Figure 26:**
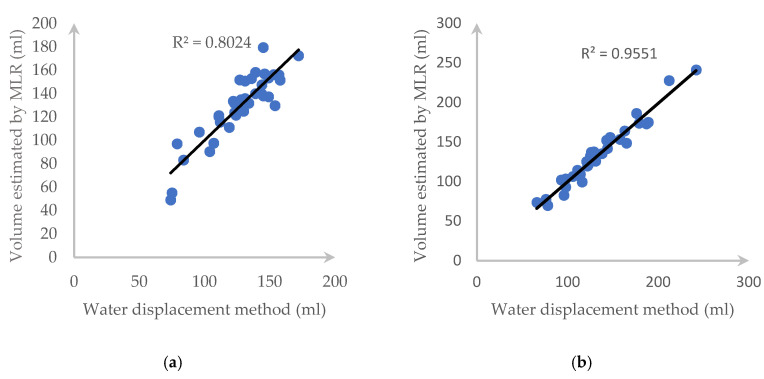
*R*^2^ and correlation between MLR and water displacement method for: (**a**) a carrot and (**b**) a cucumber.

After using the MLR, the ±1.96 standard deviation of each type of the experimental fruit is illustrated in Figure 25. Moreover, the correlation as well as *R*^2^ are also shown in Figure 26.

### 3.5. Mass Estimation

For the result of the mass estimation and classification, Section 3.5.1. and Section 3.5.2 below shows the standard illustrated in Table 3 and Table 4 as well as the confusion matrix described in Table 5 and Table 6 of the validation of carrots and cucumbers. The accuracy gained for this mass estimation is 95% for the carrot and 96.7% for the cucumber.

#### 3.5.1. Carrot Classification

Table 4 describes the confusion matrix when we apply Equation (18) with $d_{average}=1.0987\;g/\mathrm{mL}$. Our classification system achieved 95% accuracy in classification. The error may happen due to the error of the estimated volume, which is 3.421% and the average density.

#### 3.5.2. Cucumber Classification

Table 6 shows that there is only one wrongly predicted sample and the prediction error is 3.3% in the test dataset of the cucumber. The average density of the cucumber among 70 samples is $d_{average}=1.019\;g/\mathrm{mL}$. In addition, this result indicates that mass estimation via density is applicable for a cucumber.

### 3.6. Computational Expense

The computational device that we used for this research was Dell 5558 with Intel Core i7-5500U (2.4 Ghz). In addition, the OpenCV Library using Python programming language is also used in this project. After measuring the computational time with 100 repetitions, the processing time for each fruit or vegetable is in the range of 0.032 to 0.05 s. This means that we can estimate and classify the mass of 30–50 fruits/vegetables per second. Currently, we use a spatial resolution of 1538 × 2175 for our image datasets. We can safely expect that, when the resolution of images reduces to 1080 × 1440 or 720 × 960 pixels, our calculation speed will greatly increase while the calculation accuracy is still reasonable with the reduction of 0.2078% and 1%, respectively. Moreover, the time execution also reduces from 0.017 to 0.035 s for the resolution of 1080 × 1440 pixels and from 0.009 to 0.016 s for the resolution of 720 × 960 pixels. In addition, we have not fully optimized our code or made use of graphical processing units (GPU). Implementing those would also lead to faster calculation speed as well. These improvements will be implemented in the near future.

## 4. Discussion

From Table 7 and Table 8, we can see that other methods’ accuracy rates are slightly lower and have a higher deviation than our research except for that of the ANN method on estimating the volume of the carrot.

In detail, the errors for the volume estimation of the proposed method were 3.4% and 3.2% for the case of the carrot and cucumber, respectively. With the same datasets, the disk method’s error rates were 6.7% and 6.6%, while those of the ANN method were 3.9% and 5.5%, and MLR’s error rates were 8.3% and 5.4%, respectively. The standard deviation of our method on estimating the volume of the carrot ranked second, which was 7.4 mL, surpassed by ANN’s one, which was 6.2 mL, and was followed by 12.5 mL and 14.8 mL when using MLR and the disk method. However, for the standard deviation of the cucumber dataset, the highest value was 15.1 mL for the disk method, while our proposed method ranked first with 6.7 mL. This was followed by 8.4 mL of MLR and 9.1 mL of the ANN method.

In addition, most of the works that we have done are completely automatic by using Computer Vision while Örnek et al. had to measure each carrot manually [37]. Moreover, the accuracy of the disk method with two side images will decrease due to some loss at the contact surface between the object and the base. Additionally, the time execution for the disk method is also higher when each slice’s thickness is 1 pixel.

With a high correlation between mass and volume, we can estimate the weight via the density. All the results illustrated that the image processing technique can be applied in the estimation size and mass of slender axi-symmetric fruits/vegetables and gained high accuracy and was fast enough to be embedded into the production lines.

**Figure 27:**
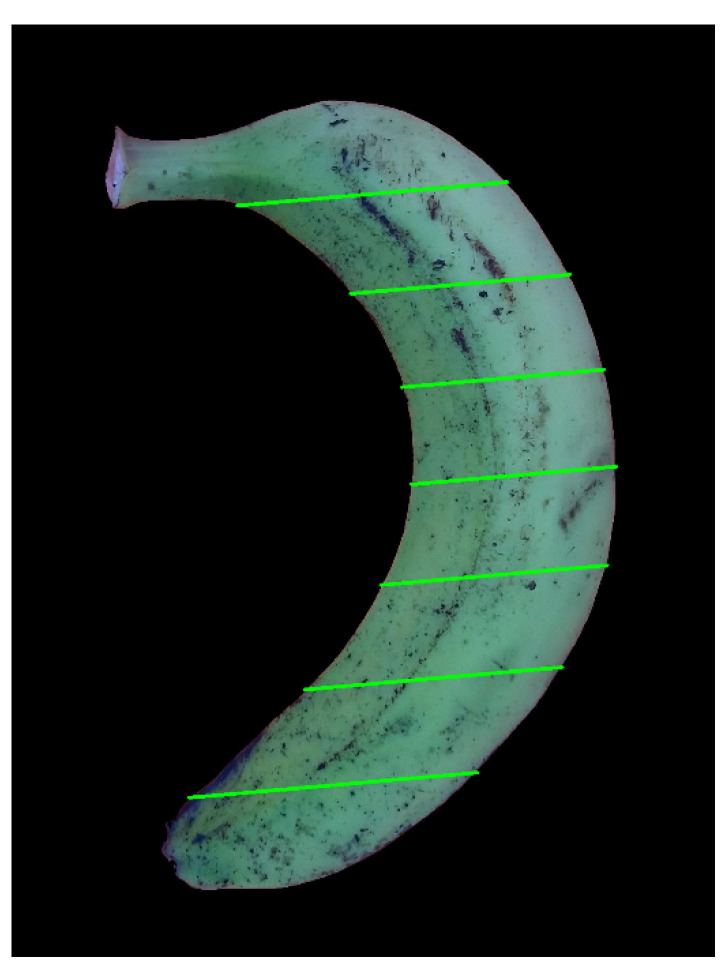
An example that our system can deal with bending fruits/vegetables.

The errors of our system come from many phases during the process such as the preprocessing step, camera, and computational rounded. The error will be higher when the fruits/vegetables in which its special shape is not axi-symmetric such as a banana [36]. However, the proposed method is able to work with a banana, which is the type of bending agricultural products shown in Figure 27. In addition, our system can even process the fruits/vegetables from different angles, which is the concern of Venkatesh et al. [19].

Figure 27 illustrated the slicing of a banana using our method. We found that the result is still reasonable with a mean error of 5.7% and the deviation of estimated volume is only 4.5875 mL. With bananas having smaller bending radii, our method provided better results. The error is only 1.2%. This demonstrates that our method is still applicable to fruits/vegetables having small bending radii. Additionally, a further improvement needs to be implemented in the near future.

## 5. Conclusions

In this paper, we proposed a method to estimate mass/volume and sizes of slender axi-symmetrical agricultural products using image processing. Experimental results show good results for products such as carrots and cucumbers. The mean of volume estimation error is 3.42% (σ = 2.60%) and 3.20% (σ = 2.82%) for the carrot and the cucumber, respectively. Furthermore, our method has a very low computational requirement. The average computing time for images having 1538 × 2175-pixels is only in the range of 30 to 50 ms, which is very favorable for real-time grading/sorting applications. Furthermore, this approach only requires one camera mounted on the top of the imaging chamber to capture one single top-view two-dimensional image. The samples do not need to be especially aligned. Therefore, they do not require any special mechanical alignment devices from the conveyor belts.

The present work may be extended in some ways.

- We can improve the main axis extraction algorithm from the top-view image for products with bending shapes. Preliminary testing on fruits with bending shapes show that the accuracy of our method reduces as the bending of the fruits becomes larger.
- The background segmentation can readily be modified for other fruits. The estimated mass/volume and size may be used together with the online defects/blemishes and color sorting. We may use another color space, such as HSI or YCbCr, for cases with difficult lighting.
- The shape classification can be implemented after the background subtraction step of our system. Referring to Table A1, we will test our datasets with the ANN, FD, and MLR classifier due to high precision in shape detection. After that, we may consider the processing time of each classifier to choose the optimal one that is suitable for our system.

## Figures and Tables

**Table 1:** The paired t-test between our proposal and water displacement.

	Sig. (2-Tailed)	95% Confidence Level of the Mean Difference	
		Lower	Upper
Carrot	*p* = 0.204	−0.3773	1.7576
Cucumber	*p* = 0.406	−0.607	1.4943

**Table 2:** R^2^ of the BP, LM, and PUNN algorithm [37].

Training Algorithm	BP	LM	PUNN
$R^{2}$	0.94281	0.95616	0.95733

**Table 3:** Carrot classification.

Type	Code	Weight (g)
Small	S	80–125
Medium	M	125–200
Large	L	200–250
Extra Large	2L	250–320

**Table 4:** Confusion matrix of mass prediction through an estimated volume.

Class	S	M	L	2L
S	24	0	0	0
M	2	37	0	1
L	0	2	33	0
2L	0	0	1	21

**Table 5:** Cucumber classification.

Type	Code	Weight (g)
Small	S	<100
Medium	M	100–150
Large	L	150–200
Extra Large	XL	>200

**Table 6:** Confusion matrix of mass prediction through an estimated volume.

Class	S	M	L	XL
S	19	1	0	0
M	0	21	1	0
L	0	0	25	1
XL	0	0	0	23

**Table 7:** Comparison between our proposed method and other methods on a carrot dataset.

Product	Method	Mean Error (%)	R^2^	Standard Deviation (mL)
Carrot	Proposed method	3.421	0.9805	7.4595
	Disk method	6.7021	0.9289	14.868
	ANN method	3.9924	0.9286	6.2846
	Using MLR	8.3444	0.8024	12.5503

**Table 8:** Comparison between our proposed method and other methods on the cucumber dataset.

Product	Method	Mean Error (%)	R^2^	Standard Deviation (mL)
Cucumber	Proposed method	3.2025	0.982	6.7081
	Disk method	6.6577	0.9199	15.1942
	ANN method	5.5339	0.9482	9.1796
	Using MLR	5.4615	0.9551	8.4656

## Abbreviations

The following abbreviations are used in Appendix A.

ANN	Artificial neural network
BP	Back projection
BPNN	Back propagation neural network
DA	Discriminant Analysis
FD	Fourier-descriptor
FDR	Fisher discrimination ratio
FNN	Feed forward neural network
FSCABC	Fitness-scaled chaotic artificial bee colony algorithm
LBP	Local binary pattern
MLR	Multiple linear regression
PCA	Principal component analysis
PNN	Probabilistic neural network
RBPNN	Radial basis probabilistic neural networks
SVM	Support vector machine
WLD	Weber local descriptor

## Appendix A

Table A1 shows the current research related to fruit classification and grading methods. Some of the literature reviews for some specific features are also summarized in this table.

**Table A1:** Summary of recent grading methods and fruit classification.

Fruit	Features	Classifier	Accuracy (%)	Reference
Date	Color, texture	Nearest Neighbor, DA, ANN	83–98	Jana et al. [44]
	Size, shape, texture,	LBP, WLD, FDR	98	Muhammad et al. [45]
	color	BP		Zhang et al. [46]
Mango	Weight, volume, size, shape	FD	98	Ibrahim et al. [15]
	Maturity, size	Fuzzy, thermal imaging	90	Pandey et al. [47]
	Size, volume	MLR, ANN	96.7	Schulze et al. [48]
	Shape, weight	FD, DA/SVM/Weight	95	Sa’ad et al. [49]
Tomato	Color, texture	PCA, SVM	92	Semary et al. [50]
	Color, shape, texture	PNN	84.4	Arjenaki et al. [51]
Orange	Color, texture	ANN	88	Capizzi et al. [52]
Apple	Color, size	Naive Bayes	91	Ronald et al. [53]
Kiwi	Shape, weight, volume	MLR	98.3	Fu et al. [54]
Strawberry	Color, size, shape	Cluster Analysis, Multidimensional scaling, DA	>68	Yamamoto et al. [55]
Cucumber	Size, shape	ANN	97	Kheiralipour et al. [56]
Pear	Size, volume	ANN, MLR	97	Forbes et al. [14]
Apple	Size, volume	ANN	99.4	Ziaratban et al. [26]
Carrot	Size, volume	ANN	99.9	Örnek et al. [37]
